# Preparation and Neuroprotective Activity of Glucuronomannan Oligosaccharides in an MPTP-Induced Parkinson’s Model

**DOI:** 10.3390/md18090438

**Published:** 2020-08-23

**Authors:** Yingjuan Liu, Weihua Jin, Zhenzhen Deng, Jing Wang, Quanbin Zhang

**Affiliations:** 1 Key Laboratory of Experimental Marine Biology, Center for Ocean Mega-Science, Institute of Oceanology, Chinese Academy of Sciences, Qingdao 266071, China; liuyjmiao@163.com (Y.L.); dengzhenzhen16@mails.ucas.ac.cn (Z.D.); jingwang@qdio.ac.cn (J.W.); 2 Laboratory for Marine Biology and Biotechnology, Qingdao National Laboratory for Marine Science and Technology, Qingdao 266071, China; 3 University of the Chinese Academy of Sciences, Beijing 100049, China; 4 College of Biotechnology and Bioengineering, Zhejiang University of Technology, Hangzhou 310014, China; jinweihua@zjut.edu.cn

**Keywords:** glucuronomannan oligosaccharides, behavioral deficits, Parkinson’ disease, apoptosis

## Abstract

Parkinson’s disease (PD), characterized by dopaminergic neuron degeneration in the substantia nigra and dopamine depletion in the striatum, affects up to 1% of the global population over 50 years of age. Our previous study found that a heteropolysaccharide from *Saccharina japonica* exhibits neuroprotective effects through antioxidative stress. In view of its high molecular weight and complex structure, we degraded the polysaccharide and subsequently obtained four oligosaccharides. In this study, we aimed to further detect the neuroprotective mechanism of the oligosaccharides. We applied MPTP (1-methyl-4-phenyl-1,2,3,6-tetrahydropyridine) to induce PD, and glucuronomannan oligosaccharides (GMn) was subsequently administered. Results showed that GMn ameliorated behavioral deficits in Parkinsonism mice. Furthermore, we observed that glucuronomannan oligosaccharides contributed to down-regulating the apoptotic signaling pathway through enhancing the expression of tyrosine hydroxylase (TH) in dopaminergic neurons. These results suggest that glucuronomannan oligosaccharides protect dopaminergic neurons from apoptosis in PD mice.

## 1. Introduction

As one of the most common neurodegenerative disorders, the incidence of Parkinson’s disease (PD) affects up to 1% of the global population over 50 years of age [1,2,3]. PD patients often suffer from progressive cognitive and motor deficits, such as tremors, bradykinesia and rigidity, primarily due to dopaminergic neuron degeneration in the substantia nigra (SN) and dopamine (DA) depletion in the striatum [4,5]. Over the past decades, levodopa has been established as a gold standard agent for the treatment of PD. It has dramatically inhibited the mortality rate of PD patients at early stages of the disease; however, clinical surveys demonstrate that the longevity of PD patients, with oral drug taking compliance, is still decreased [2,6]. Although many factors are thought to contribute to the pathogenesis of PD, such as aging, neuroinflammation, mitochondrial dysfunction, oxidative stress, and apoptosis, the pathogenesis underlying the lesions remains indeterminate [7,8,9]. Hence, further investigation of promising neuroprotective approaches against PD remains urgently needed [10].

Recently, several studies indicated that bioactive polysaccharides or oligosaccharides from animals, plants, or fungi presented potential effective treatment in the prevention of development of neurodegenerative disorders [11,12,13]. Li and colleagues found that *Astragalus* polysaccharides suppressed 6-hydroxydopamine-induced neurotoxicity in *Caenor-habditis elegans* [14], and Kim and colleagues found that fucoidan attenuated 6-hydroxydopamine-induced neurotoxicity by exerting anti-oxidative and anti-apoptotic effects in SH-SY5Y cells [15]. Jiang and colleagues demonstrated that oligomannurarate 971 derived from a marine plant exhibits neuroprotective effects against Aβ peptide toxicity [16]. The brown seaweed, *Saccharina japonica*, which contains various bioactive compounds such as iodine, mannitol, proteins, minerals, and polysaccharides, is a common food in East Asia [17]. Recent studies have demonstrated that polysaccharides from *Saccharina japonica* show great potential in anti-apoptosis, antioxidant, anticoagulant, antitumor, antiviral and anti-inflammatory activity [18]. Our previous study found that the sulfated heteropolysaccharides from *Saccharina japonica* protected SH-SY5Y cells from H_2_O_2_-induced apoptosis by affecting the PI3K/Akt signaling pathway [19]. Considering its high molecular weight and complex structure, this sulfated heteropolysaccharide was hydrolyzed in dilute sulfuric acid, and a series of glucuronomannan oligosaccharides (GMns) were prepared, with their potential role in resistance against PD subsequently investigated in a PD model. MPTP (1-methyl-4-phenyl-1,2,3,6-tetrahydropyridine), a lipophilic compound, selectively damages neurons in the nigrostriatal dopaminergic pathway and causes Parkinsonism. The C57BL/6 mouse strain is known to be highly susceptible to this neurotoxin, and MPTP-treated mice have been widely used as an in vivo model to test therapeutic strategies in PD [3].

## 2. Results

### 2.1. Characterization of Glucuronomannan Oligosaccharides

**Figure 1:**
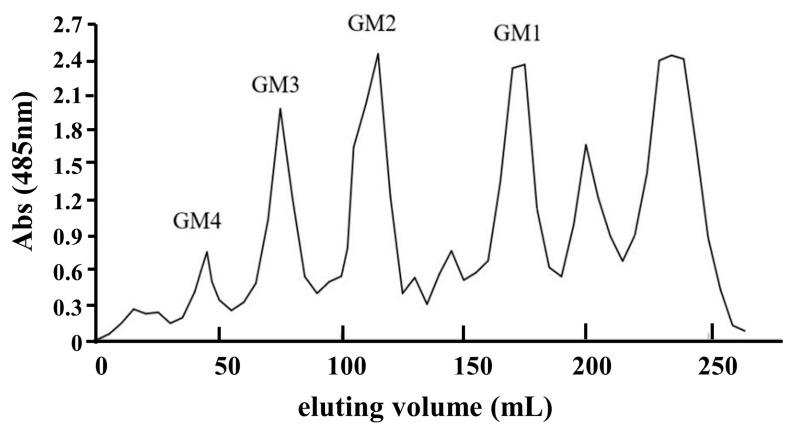
Preparation of glucuronomannan oligosaccharides (GMn) by gel filtration chromatography on a Bio-Gel P-4 Gel column (extra fine, <45 μM; 2.6 cm × 100 cm) eluted with 0.5 M NH4HCO3 at a flow rate 0.14 mL min^−1^.

Preparation of glucuronomannan oligosaccharides was described in our previous study [20]. Briefly, crude fucoidan was degraded in ascorbic acid (0.5% (g/mL)) and 0.3% hydrogen dioxide (mL/mL) for 1 h at room temperature. After ultrafiltration, concentration and precipitation by ethanol, degraded polysaccharides underwent anion exchange chromatography on a DEAE-Bio Gel Agarose FF gel (12 cm × 70 cm) with elution by 0.5 M (35L) (F0.5), 1 M (35L) (F1) and 2 M NaCl (35L) (F2). Finally, the F0.5, F1 and F2 factions were dried using an infrared lamp. The sulfated heteropolysaccharide fraction F0.5 was degraded by dilute sulfuric acid and fractionated using activated carbon column chromatography. Fraction Y2 was obtained from the 50% ethanol eluent, and further fractionated by gel filtration chromatography on a Bio-Gel P-4 Gel column to yield six fractions (Figure 1). Monosaccharide constitution analysis indicated that fractions GM1, GM2, GM3 and GM4 represented a series of glucuronomannan oligosaccharides consisting of glucuronic acid and mannose with a molar ratio of 1:1. Results of HPLC analysis confirmed that GM1, GM2, GM3 and GM4 were relatively pure. Their structures were previously elucidated [21,22]. These glucuronomannan oligosaccharides were composed of a repeating disaccharide unit of 4-linked gluconic acid (GlcA) and 2-linked mannose (Man). The degrees of polymerization (DP) of GM1, GM2, GM3 and GM4 were 2, 4, 6 and 8, respectively.

Considering that the yield of GM4 (the glucuronomannan tetramer) was quite low, the neuroprotective activities of GM1, GM2 and GM3 were investigated in an MPTP-induced PD mouse model.

### 2.2. Effects of GMn on Behavioral Deficits in PD Mice

**Figure 2:**
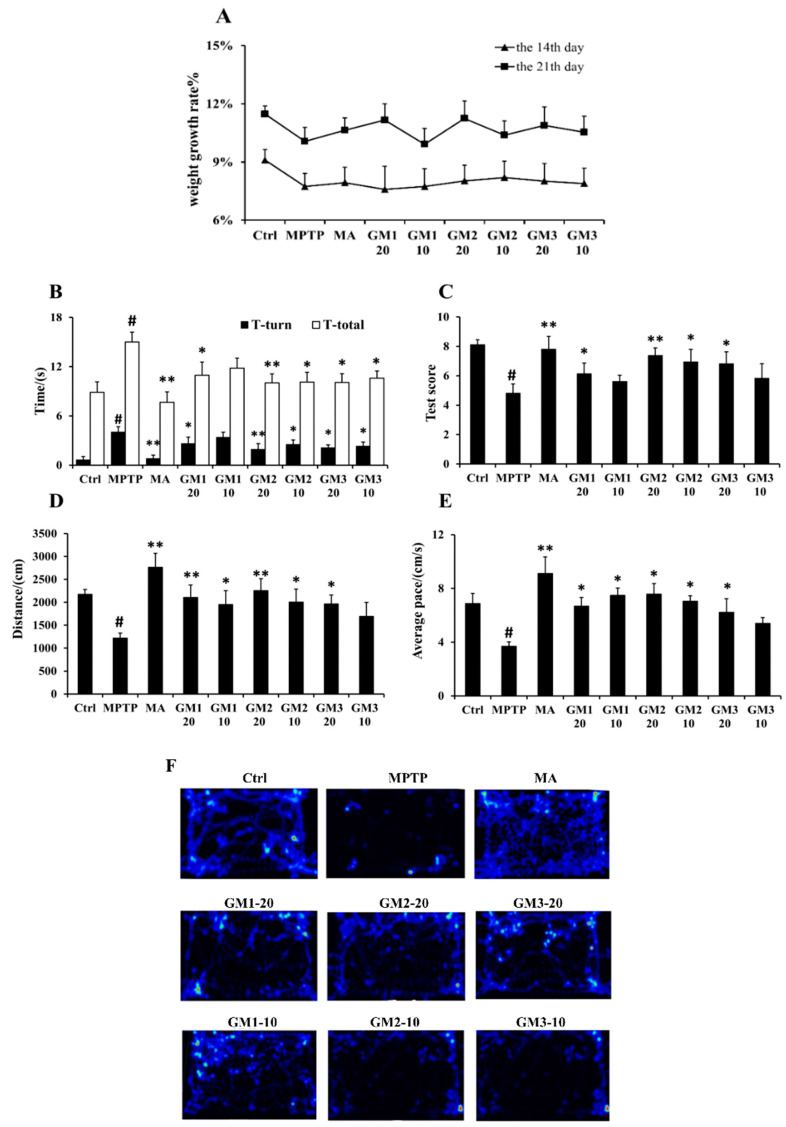
Effects of GMn on behavioral deficits in PD mice. (**A**) The weight growth rate. (**B**) T-turn and T-total of pole test. (**C**) Traction performance. (**D**) Distance of open-field performance. (**E**) Mean velocity of open-field performance. (**F**) Activity heat map of the mice. Ctrl: the control group; MPTP: the mice treated with 20 mg·kg^−1^ MPTP alone; MA: the mice treated with Madopar (70 mg·kg^−1^) followed by MPTP. GM1/2/3-10: the mice treated with 10 mg·kg^−1^; GM1/2/3-20: the mice treated with 20 mg·kg^−1^; # *P* < 0.05 compared with the control group; * *P* < 0.05 or ** *P* < 0.01compared with MPTP group. The values are given as mean ± SEM (*n* = 6).

In our previous study, we detected the structure–activity relationship of sulfated hetero/galactofucan polysaccharides on dopaminergic neuron in MPTP-induced PD mice [23]. We found that treatment with low molecular weight fucoidan (DF) and its fractions DF1 and DF2 at 25 mg/mL clearly prevented a decrease in the content of DA and improved the protein level of TH. GMn was the backbone of DF. GMn has a small molecular weight and a simple structure, which is more favorable for interaction with cells. Thus, we chose two doses (10 mg/mL and 20 mg/mL) to detect the neuroprotective effects. In this study, MPTP was applied to induce a PD model in mice. Madopar (MA) was applied as the positive control to evaluate the protective effects of GMn. There was no significant change in weight (Figure 2A). Pole test revealed that MPTP markedly impaired performance of the mice (Figure 2B). GMn administration markedly alleviated this behavioral disorder, particularly GM2, which showed stronger effects. Furthermore, we applied a traction test to evaluate muscular strength. Figure 2C showed that injection of MPTP attenuated physical coordination, but this impairment was markedly improved by treatment with GM1, GM2 and GM3. Similar to previous observation, GM2 significantly enhanced muscular strength in mice. In addition, the locomotor activity of mice was measured with an open field test (Figure 2D–E). MPTP resulted in a significant decrease in total distance and mean velocity, while GM1/2/3 effectively enhanced spontaneous activity in PD mice, improving locomotor abilities. These results were in accordance with those observed in Figure 2F (activity heat map), in which GMn improved behavioral deficits. In particular, GM2 at a dose of 20 mg/kg presented the greatest effect in improving behavioral deficits in PD mice. However, no significant difference was observed among GM1, GM2 and GM3. MA treatment offers levodopa, the precursor of dopamine, and has been widely used as a clinical drug in patients with PD. After MPTP treatment, the levels of dopamine in the striatum were significantly decreased, resulting in behavioral disorders. When treated with Madopar, a large amount of dopamine was added over time, leading to a transient state of arousal in mice; thus, a high increase in the MA-treated group was observed compared to the control group.

### 2.3. Effects of GMn on Levels of DA, 5-hydroxytryptamine (5-HT) and Their Metabolites

**Figure 3:**
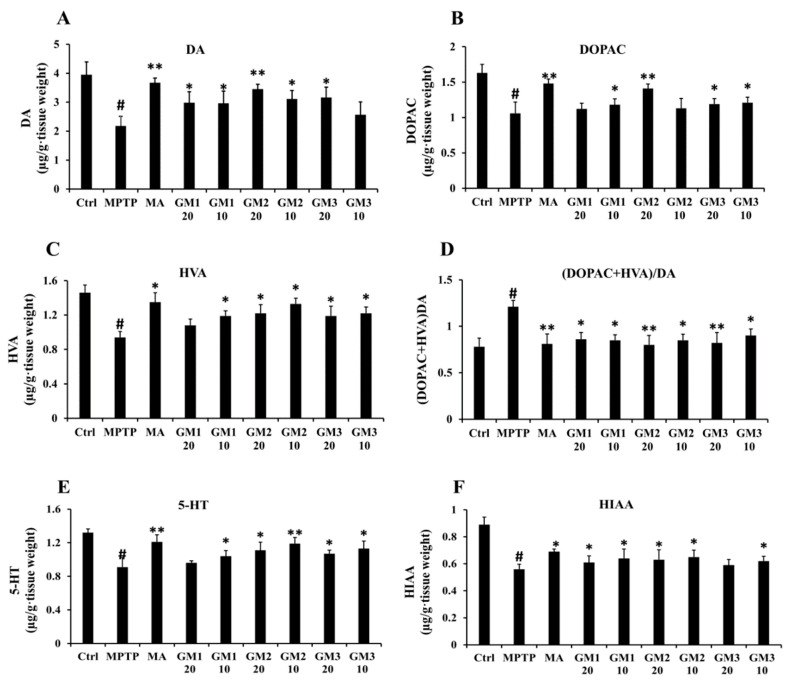
Effects of GMn on the levels of DA, 5-HT and their metabolites. (**A**) The level of DA. (**B**) The level of DOPAC. (**C**) The level of HVA. (**D**) The ratio of (DOPAC + HVA)/DA. (**E**) The level of 5-HT. (**F**) The level of HIAA. Ctrl: the control group; MPTP: the mice treated with 20 mg·kg^−1^ MPTP alone; MA: the mice treated with Madopar (70 mg·kg^−1^) followed by MPTP. GM1/2/3-10: the mice treated with 10 mg·kg^−1^; GM1/2/3-20: the mice treated with 20 mg·kg^−1^; # *P* < 0.01 compared with the control group; * *P* < 0.05 or ** *P* < 0.01 compared with MPTP group. The values are given as mean ± SEM (*n* = 4).

As shown in Figure 3A–D, the neurotoxicity of MPTP was verified by a decrease in the striatal content of DA and its metabolites as measured by HPLC-ECD. Treatment with GMn clearly prevented a decrease in the content and effectively reversed the abnormally increased ratio of (dihydroxy-phenyl acetic acid (DOPAC) + homovanillic acid (HVA))/DA induced by MPTP (*P* < 0.05), implying that GMn significantly inhibited the turnover of DA in the striatum. Meanwhile, GMn inhibited the decrease in 5-HT and its metabolite hydroxyindoleacetic acid (HIAA) lesions caused by administration of MPTP (*P* < 0.05) (Figure 3E–F). While the protective effect of GM2 on DA, 5-HT and their metabolites was more obvious, there was no significant difference among GM1, GM2 and GM3. Therefore, we speculated that GM1, GM2 and GM3 inhibit PD progression as a form of complex oligosaccharides, GMn.

### 2.4. Effects of GMn on Expression of monoamine oxidase B (MAO-B) and α-synuclein

**Figure 4:**
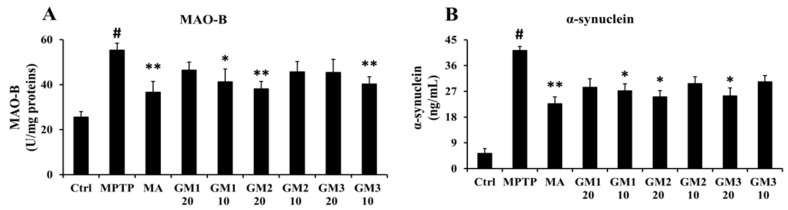
Effects of GMn on the expression of MAO-B and α-synuclein. (**A**) The level of MAO-B. (**B**) The level of α-synuclein. # *P* < 0.01 compared with the control group; Ctrl: the control group; MPTP: the mice treated with 20 mg·kg^−1^ MPTP alone; MA: the mice treated with Madopar (70 mg·kg^−1^) followed by MPTP. GM1/2/3-10: the mice treated with 10 mg·kg^−1^; GM1/2/3-20: the mice treated with 20 mg·kg^−1^; # *P* < 0.01 compared with the control group; * *P* < 0.05 or ** *P* < 0.01 compared with MPTP group. The values are given as mean ± SEM (*n* = 3).

As shown in Figure 4A, MPTP treatment induced a marked increase in expression of MAO-B compared to the control group. MAO-B catalyzes the oxidative deamination of dopamine to yield hydroperoxide, participating in Fenton-type reactions with Fe (II) to generate reactive oxygen species, which is consistent with results that MPTP decreases the levels of DA. However, GM1/2/3 inhibited this trend induced by MPTP, especially GM2 at 20 mg/kg, GM1 at 10 mg/kg and GM3 at 10 mg/kg, notably suppressing the activity of MAO-B. A-synuclein, a protein specific to neurons indirectly involved in neurotransmitter release, was detected in this study, as shown in Figure 4B. Compared to the control group, a significant increase was observed after MPTP treatment; however, GM2 and GM3 at 20 mg/kg and GM1at 10 mg/kg significantly decreased levels of α-synuclein (*P* < 0.05). These results revealed that GMn inhibits the expression of MAO-B and α-synuclein to resist damage from MPTP.

### 2.5. Effects of GMn on the Expression of TH and dopamine transporter (DAT) Loss in the Striatum

**Figure 5:**
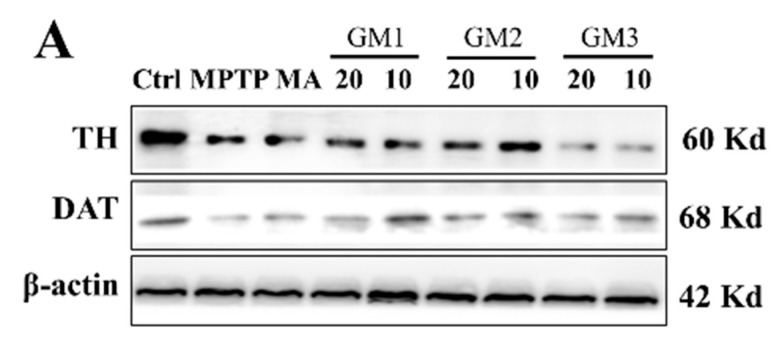

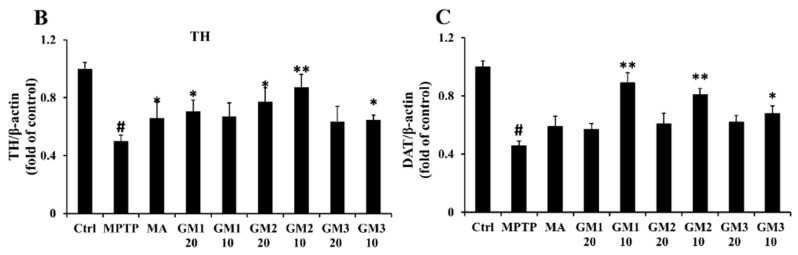
Effects of GMn on the expression of TH and DAT loss in striatum. (**A**) The original bands of TH and DAT. (**B**) Quantification of TH. (**C**) Quantification of TH. Ctrl: the control group; MPTP: the mice treated with 20 mg·kg^−1^ MPTP alone; MA: the mice treated with Madopar (70 mg·kg^−1^) followed by MPTP. GM1/2/3-10: the mice treated with 10 mg·kg^−1^; GM1/2/3-20: the mice treated with 20 mg·kg^−1^; # *P* < 0.01 compared with the control group; * *P* < 0.05 or ** *P* < 0.01 compared with MPTP group. The values are given as mean ± SEM (*n* = 3).

The protective roles of GM1/2/3 in PD mice led us to hypothesize that GM1/2/3 might protect dopaminergic neurons from MPTP-induced apoptosis. Therefore, we applied Western blot analysis to TH and DAT, as dopaminergic neurons are the main tissue that produces TH in vivo. Results revealed that MPTP induced significant decreases in the expression of DAT and TH (Figure 5), while GM1/2/3 significantly restrained this decrease. Notably, GM2 showed a more significant effect on the improvement of TH. Furthermore, GM1, GM2 and GM3 at 10 mg/kg markedly enhanced protein expression of DAT. Levels of DA and its metabolites (DOPAC and HVA) were decreased in the striatum after MPTP treatment due to impairment of dopaminergic neurons; however, GMn markedly reversed this trend, indicated by the decreased ratio of (DOPAC + HVA)/DA, which was consistent with the results that GMn inhibited apoptosis and prevented TH loss. This implied that GMn inhibits turnover of dopamine in the striatum of MPTP mice. Additionally, MA as the positive control showed a less marked effect than GMn on expression of TH and DAT.

### 2.6. Effects of GMn on Apoptosis-Related Proteins

**Figure 6:**
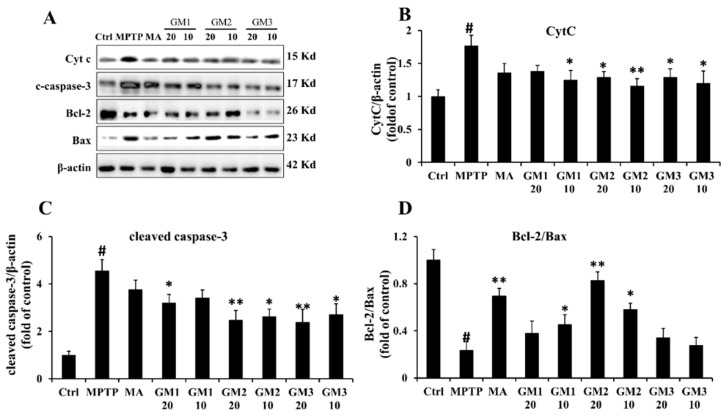
Effects of GMn on the apoptosis-related proteins. (**A**) The original bands of Cytochrome c (Cyt c), cleaved caspased-3, Bcl-2 and Bax. (**B**) Quantification of Cyt c. (**C**) Quantification of caspase-3. (**D**) The ratio of Bcl-2/Bax. Ctrl: the control group; MPTP: the mice treated with 20 mg·kg^−1^ MPTP alone; MA: the mice treated with Madopar (70 mg·kg^−1^) followed by MPTP. GM1/2/3-10: the mice treated with 10 mg·kg^−1^; GM1/2/3-20: the mice treated with 20 mg·kg^−1^; # *P* < 0.01 compared with the control group; * *P* < 0.05 or ** *P* < 0.01compared with MPTP group. The values are given as mean ± SEM (n = 3).

The mitochondrial apoptosis pathway is involved mainly in the apoptosis of dopaminergic neuronal cells. Therefore, we applied Western blot analysis to examine the expression of Bax, Bc-2 and cytochrome c. Figure 6A–C demonstrates that MPTP increased the release of cytochrome c and activation of caspase-3, while treatment with GM1/2/3 prevented the increased trend in cytochrome c and caspase-3. Furthermore, MPTP increased expression of Bax and decreased expression of Bcl-2, resulting in a decrease in the Bcl-2/Bax ratio, whereas administration of GM1/2/3 markedly increased the ratio (Figure 6D). Specifically, GM2 at 20 mg/kg presented the best effect on the ratio. These results imply that GM1/2/3 inhibits mitochondrial apoptosis to prevent cell injury. MA did not effectively inhibit apoptosis induced by MPTP.

### 2.7. Effects of GMn on Levels of Nerve Growth Factor (NGF) and Receptor Tyrosine Kinase (TrkA)

**Figure 7:**
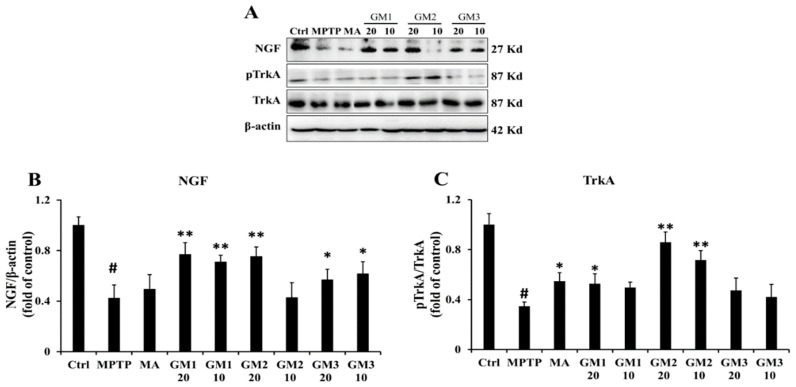
Effects of GMn on the level of NGF and TrkA. (**A**) The original bands of NGF, TrkA and TrkA. (**B**) Quantification of NGF. (**C**) Quantification of TrkA. Ctrl: the control group; MPTP: the mice treated with 20 mg·kg^−1^ MPTP alone; MA: the mice treated with Madopar (70 mg·kg^−1^) followed by MPTP. GM1/2/3-10: the mice treated with 10 mg·kg^−1^; GM1/2/3-20: the mice treated with 20 mg·kg^−1^; # *P* < 0.05 versus the control group; * *P* < 0.05 or ** *P* < 0.01 versus MPTP group. The values are given as mean ± SEM (*n* = 5).

To determine whether GMn treatment promotes the survival of neurons, we tested the effectiveness of a representative neurotrophin, NGF, against MPTP-induced damage. As shown in Figure 7, MPTP treatment led to the consumption of NGF and low expression of pTrkA (*P* < 0.01). However, GM1/2/3 elevated expression of NGF. In particular, GM2 exerted a stronger protective effect on the improvement of NGF and TrkA than MA. Nerve growth factor (NGF), an evolutionary conserved factor with numerous functions, stimulates signals through receptor tyrosine kinases (TrkA).

## 3. Discussion

Animal models that recreate specific pathogenic events and their behavioral outcomes have been used to simulate neurological disorders in humans and provide an indispensable tool for basic research as a prerequisite to their testing in patients [23]. The functional similarities between C57BL/6 and humans make mice a reasonable option for the development of the clinical predictability of responses observed in preclinical investigations [24,25,26]. MPTP is transformed into its toxic derivative, 1-methyl-4-phenylpyridinium (MPP^+^), by the enzyme MAO-B in astrocytes [27,28,29]. MPP^+^ is released from astrocytes and transported into dopaminergic neurons, where it interferes with complex I, compromises adenosine triphosphate (ATP) synthesis, and destroys mitochondria. Thus, MPTP is widely used for producing animal models of Parkinson disease. In this study, we applied MPTP to induce a PD model in vivo.

Behavioral functions are reported to be sensitive in detecting functional impairments to quantify the potential efficacy of treatments designed to prevent dopamine loss [30,31]. The ability to balance and coordinate is widely used as a measure of motor skill. When administered in mice, MPTP decreases behavioral functions and striatal DA levels [32,33]. In the present study, mice treated with MPTP showed a reduction in spontaneous activities, balance vestibular integrity and muscular coordination compared to the control group, as evidenced by the open field test, pole test and traction test. However, mice were protected from MPTP toxicity due to administration of GMn, suggesting that GMn helps mice retain their natural activity and proper muscular coordination. The core pathological feature of PD is the accelerated loss of dopaminergic neurons in the substantia nigra pars compacta (SNpc) and subsequent striatal DA deficiency [2,34]. Of course, levels of DA and its metabolites (DOPAC and HVA) were decreased in the striatum after MPTP treatment due to the impairment of dopaminergic neurons; however, GMn markedly reversed this trend, as indicated by the decreased ratio of (DOPAC + HVA)/DA. These findings imply that GMn inhibits dopamine turnover in the striatum of MPTP mice.

In PD patients, apoptosis might be involved in the degeneration of dopaminergic neurons [24,35]. Thus, we hypothesized that GMn might play a beneficial role in PD by blocking neuron apoptosis. Statistical results showed that treatment with GMn suppressed apoptosis proteins in MPTP-treated mice. TH, the rate-limiting enzyme in the synthesis of DA, is often used as a marker for the integrity of dopaminergic neurons [36,37]. Previous studies have reported that MPTP reduces levels of TH [38,39], which was consistent with our results that MPTP induced a decrease in TH expression. However, treatment with GMn markedly prevented TH loss, improved DAT expression and inhibited hypercatabolism of DA. To further investigate whether GMn protects against MPTP-induced cytotoxicity, we detected changes of many signaling proteins and molecules. NGF and its high affinity TrkA receptors selectively bind and promote survival and induction of neurite outgrowth during development or various other conditions [40,41]. In our study, GMn effectively prevented the MPTP-induced decrease in NGF and the inactivated forms of TrkA.

Madopar is widely used as a “gold standard” in PD treatment. It alleviates most of the symptoms of PD by replenishing striatal DA after oral administration of the DA precursor levodopa [2]. However, many clinical surveys have found that the majority of patients develop involuntary motor response oscillations in response to this treatment, which significantly impairs their quality of life. Sadly, adverse reactions to Madopar have still not been resolved [42]. As demonstrated in our study, treatment with Madopar resulted in excessive activity in mice. However, apoptosis was not obviously inhibited in this condition. In contrast, GM1/2/3 showed weaker effects on the behavioral disorders of PD mice than MA, but GM1/2/3 significantly inhibited the occurrence of apoptosis, increased expression of NGF and its receptors, and enhanced proteins levels of TH and DAT.

The glucuronomannan oligosaccharides from *Saccharina japonica,* with a backbone of alternating 2- mannose and 4- glucuronic acid, exhibited promising neuroprotective effects in PD mice. Although GM2 presented slightly better results than GM1 and GM3, there was no marked difference observed. Taking into account the complexity of separation and purification of GM1, GM2 and GM3, we thought that GM1, GM2 and GM3 could be used as PD agents in the form of mixed complexes.

## 4. Materials and Methods

### 4.1. Chemicals

Madopar (MA) was obtained from Shanghai Roche Pharmaceuticals Ltd. (Shanghai, China). Dopamine (DA), 3, 4-dihydroxyphenylacetic acid (DOPAC), homovanillic acid (HVA), 5-hydroxytryptamine (5-HT), 5-hydroxyindoleacetic acid (HIAA) and MPTP, were purchased from Sigma-Aldrich (St. Louis, MO, USA). BCA proteins determining kit and MAO-B and α-synuclein detection kits were purchased from Beyotime (Nanjing, China). PVDF transfer membrane was purchased from Millipore Corp (Bedford, MA, USA). Rabbit antibodies to NGF, TH, DAT, pTrkA, TrkA, Bax, Bcl-2, cleaved-caspase-3, Cyt c and β-actin were obtained from Cell Signal Transduction, Cincinnati, USA.

### 4.2. Preparation of Glucuronomannan Oligosaccharides (GMns)

Glucuronomannan oligosaccharides (GMns) were prepared according to a protocol reported in a previous study with minor modifications [20]. Briefly, crude fucoidan from *Saccharina japonica* was hydrolyzed by oxidative degradation by combination with hydrogen peroxide and ascorbic acid (30 mM). The sulfated heteropolysaccharide fraction F0.5 was prepared from low molecular weight fucoidan using a DEAE-Bio Gel Agarose FF column chromatography. F0.5 was dissolved in 4% dilute sulfuric acid to a final concentration of 60 mg/mL, degraded at 100 °C for 5 h, cooled to room temperature, and then neutralized with barium hydroxide. After centrifugation, the supernatant was concentrated and fractionated on an activated carbon column chromatography. Fraction Y1 was obtained from eluent with water and Fraction Y2 was from eluent with 50% ethanol. Y2 was further separated on a Bio-Gel P-4 Gel column eluted with 0.5 M NH_4_HCO_3_ solution. The elution was assayed by a phenol–sulfuric acid method, and six main fractions were collected and lyophilized.

The molar ratio of monosaccharides composition was determined following the methods of Zhang et al. (Zhang, Zhang, Wang, Shi and Zhang, 2009). The purity of oligosaccharides was analyzed by HPLC with an ELSD detector, performed on a “click” maltose column (10 μm, 10 × 150 mm) according to the method of Jin et al. [21].

### 4.3. Animals and Drug Administration

This study was approved by the Animal Care and Ethics Committee of the Affiliated Hospital of Qingdao University in compliance with the Principles of Laboratory Animal Care that were developed by the National society for Medical Research. The Laboratory animal permit was SYXK(Lu) 2015 0003. The date of approval was 27 Feb 2019. Animals were housed with free access to food and tap water and under standard conditions, with controlled temperature (21–25 °C) and a 12 h light–dark cycle. At the end of treatment, all mice were euthanized. No mice were excluded from statistical analysis. The data analyst was blinded to treatment groups.

**Figure 8:**
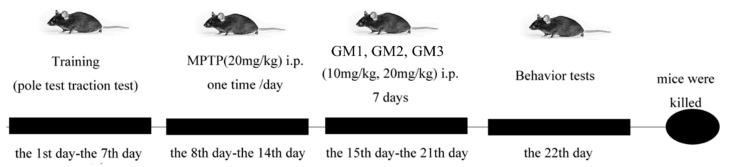
Schematic of experimental of procedure.

Adult male C57BL/6 mice (8–10 weeks, weighing 25–30 g) were randomly assigned into 9 groups with 12 animals in each group: control; MPTP only; MPTP + Madopar (70 mg·kg^−1^); MPTP + GM1/2/3 (10 mg·kg^−1^); MPTP + GM1/2/3 (20 mg·kg^−1^). There was a period of seven days for animals to acclimatize before treatments. Mice received MPTP hydrochloride intraperitoneally at a dose of 20 mg·kg^−1^ ·day^−1^ for 7 days. The control group received the same volume of saline. Thereafter, GM1/2/3 and Madopar groups were treated intraperitoneally with GM1/2/3 or Madopar for another 7 consecutive days, while the control and MPTP groups received the equivalent volume of saline. The experimental design is illustrated in Figure 8.

### 4.4. Behavioral Evaluation

Trained observers who were blinded to the treatment groups performed all behavioral experiments between 10:00 and 15:00 in a weakly-lit and quiet environment. Behavioral assessments, including the pole test, the traction test, and open-field tests, are frequently used to determine Parkinsonism-related outcomes.

#### 4.4.1. Open-Field Test

Open field testing was conducted in a white plastic arena (44 cm × 44 cm × 32 cm) with weak overhead illumination (40 W). During the testing, the behavior of the mice was monitored using a video camera that was mounted above. The EthVision software (EthVision XT, Noldus (Beijing) Information Technology Co. Ltd, Beijing, China) sampled the travelled distance, mean velocity (*V*mean), and activity heat.

#### 4.4.2. Pole Test

The pole test was performed according to previous study [43]. In brief, mice were placed head upward on the top of a rough surfaced pole (1 cm in diameter and 50 cm in height). The pole was wrapped with gauze to prevent slipping. Each mouse was allowed to descend the pole three times in a single session. The time until the mouse had turned completely downward (T-turn) and the time until it climbed down to the floor (T-total) were measured.

#### 4.4.3. Traction Test

The traction test was used to detect limb impairment, as previously described [44]. In brief, the mouse was hung by the forepaws on a horizontal wire that was 50 cm above the ground. The mouse was given a score of 3 for grasping the wire with both hind paws, 2 for grasping the wire with one hind paw, and 1 for not grasping the wire with either hind paw. The duration of suspension was recorded.

### 4.5. Western Blot Analysis

Western blot analysis procedures were performed as previously described [45]. Striatum tissues were homogenized in ice-cold lysis buffers (50 mM Tris pH7.4, 150 mM NaCl, 1% Triton X-100, 1% sodium deoxycholate, 0.1% SDS, sodium orthovanadate, sodium fluoride, EDTA and leupeptin) (50 mL•g^−1^ tissue) and centrifuged at 12,000 rpm for 5 min at 4 °C. Proteins were quantified using a BCA assay kit and separated using 12–15% SDS-PAGE gels and transferred to a PVDF membrane (Millipore, Burlington, VT, USA). Membranes were sealed with 5% bovine serum albumin for 2 h, then hybridized with primary antibodies overnight at 4 °C. Following this, samples were rinsed three times with TBST for 10 min each, and the horseradish peroxidase-conjugated IgG secondary antibody (1:2000) was added. Enhanced chemiluminescence was applied to detect the protein bands.

### 4.6. Quantification of DA, 5-HT and Their Metabolites

Striatum tissues were processed and stored at −80 °C. Content of DA, 5-HT and their metabolites was determined using an HPLC apparatus with an electro-chemical detector. Briefly, the tissues were homogenized in 0.2 M cold perchloric acid. After placed in an ice bath for 60 min, the sample was centrifuged at 12,000× g for 20 min at 4 °C, and the supernatant was transferred to a clean tube, to which 0.3 M potassium dihydrogen phosphate, 0.02 M potassium citrate, and 0.002 M Na_2_EDTA were added. After mixture and incubation in an ice bath for another 60 min, the mixture was centrifuged at 12,000× *g* for 20 min at 4 °C. The supernatant was filtered through a 0.22 μm Millipore filter and then injected into the HPLC system for analysis. The mobile phase was constituted of 0.125 M sodium citrate buffer containing 20% methanol, 0.1 mM Na_2_EDTA and 0.5 mM 1-octanesulfonic acid sodium salt adjusted to pH 4.3.

### 4.7. Assay of MAO-B and α-synuclein

The levels of MAO-B and α-synuclein in the striatum was measured using sandwich enzyme-linked immunosorbent assay and commercial kit according to the manufacturer’s instructions. The absorbance of samples was read at 450 nm by a microplate reader (BioTek, USA) and demarcated according to related standard curves.

### 4.8. Statistical Analysis

The data and statistical analyses comply with the recommendations on experimental design and analysis in pharmacology. Data analysis was performed using SPSS software (SPSS 17.0, SPSS Inc., Chicago, IL, USA). One-way analysis of variance (ANOVA) was used to compare the mean differences among the groups. The results are shown as the mean ± SEM and a level of *P* ≤ 0.05 was considered to be statistically significant.

## 5. Conclusions

In this study, four glucuronomannan oligosaccharides monomers with different degrees of polymerization were prepared from the heteropolysaccharides. We applied three GMn to investigate their potential neuroprotective effects in a PD model. Results demonstrated that GMn attenuated MPTP-induced behavioral deficits via prevention of dopaminergic neuron loss in PD mice. These results suggest that GMn might present promising agents for PD and pave the way for exploration of their clinical application in the future.
